# Utilization of Spent Coffee Grounds as a Food By-Product to Produce Edible Films Based on κ-Carrageenan with Biodegradable and Active Properties

**DOI:** 10.3390/foods13121833

**Published:** 2024-06-11

**Authors:** Dani Dordevic, Natalia Gablo, Lenka Zelenkova, Simona Dordevic, Bohuslava Tremlova

**Affiliations:** Department of Plant Origin Food Sciences, Faculty of Veterinary Hygiene and Ecology, University of Veterinary Sciences Brno, Palackeho tr. 1946/1, 612 42 Brno, Czech Republic; dordevicd@vfu.cz (D.D.); h20176@vfu.cz (L.Z.); jancikovas@vfu.cz (S.D.); tremlovab@vfu.cz (B.T.)

**Keywords:** food waste valorization, antioxidant properties, polyphenols, antioxidant, sustainable packaging

## Abstract

Coffee ranks as the second most consumed beverage globally, and its popularity is associated with the growing accumulation of spent coffee grounds (SCG), a by-product that, if not managed properly, constitutes a serious ecological problem. Analyses of SCG have repeatedly shown that they are a source of substances with antioxidant and antimicrobial properties. In this study, we assessed SCG as a substrate for the production of edible/biodegradable films. The κ-carrageenan was utilized as a base polymer and the emulsified SCG oil as a filler. The oil pressed from a blend of Robusta and Arabica coffee had the best quality and the highest antioxidant properties; therefore, it was used for film production. The film-forming solution was prepared by dissolving κ-carrageenan in distilled water at 50 °C, adding the emulsified SCG oil, and homogenizing. This solution was cast onto Petri dishes and dried at room temperature. Chemical characterization showed that SCG increased the level of polyphenols in the films and the antioxidant properties, according to the CUPRAC assay (CC1 23.90 ± 1.23 µmol/g). SCG performed as a good plasticizer for κ-carrageenan and enhanced the elongation at the break of the films, compared with the control samples. The solubility of all SCG films reached 100%, indicating their biodegradability and edibility. Our results support the application of SCG as an active and easily accessible compound for the food packaging industry.

## 1. Introduction

Undoubtedly, the invention of plastic is considered a civilizational breakthrough that has significantly influenced lifestyle and permeated all aspects of modern society. However, this advancement comes with serious environmental problems. Plastic production has soared since the 1950s when annual production averaged 1.5 million metric tons. By 2021, global plastics production had reached a staggering 390 million metric tons. This massive increase has led to widespread environmental pollution, including ocean pollution, landfill accumulation, micro plastic contamination, and consequently greenhouse gas emissions [1].

Due to plastic’s favorable properties, including its durability, strength, simplicity in manufacturing, and low cost of production, it was adopted to a large extent in various industries, including the food industry. The food packaging sector is responsible for almost half of the world’s plastic production, as it enables the development of convenient packaging systems that can be adapted to any type of food. Plastic is unquestionably attractive as a packaging material, but its heavy use causes serious environmental problems. A comprehensive assessment of the global trade in plastic packaging has shown that a large proportion of plastic packaging is used only once [2]. Approximately 71% of this non-biodegradable waste ends up in landfills or water streams, ending up in the oceans as microparticles, and only 9% of plastic has ever been recycled [3].

Coffee ranks as the second most frequently consumed beverage in the world and belongs to the most traded agricultural commodities across the world [4,5]. To illustrate the situation better, close to 170 million 60-kilogram bags of coffee were consumed in 2020–2021, and the demand continues to grow. The popularity of coffee translates into the generation of considerable quantities of by-products, such as spent coffee grounds [6,7,8]. 

Considering the growing coffee industry and the increasing trend in the coffee market, this will result in an even greater accumulation of coffee-by-products waste. According to available data, each gram of coffee bean produces about 0.91 g of SCG, meaning that SCG are the most abundant waste generated in coffee beverage preparation, and at least 60 million tons of SCG are accumulated annually [9,10,11]. SCG are noticeable waste that can cause worsening environmental pollution if not properly managed. Although a significant proportion of SCG is usually incinerated or landfilled, these materials can be used in a variety of industrial applications. The repurposing of spent coffee grounds as alternatives to petroleum-based plastic films is discussed in the food packaging sector. After the coffee brewing process, the resulting SCG are solid wet residues (61% moisture), which still have enormous advantages as they are rich in organic compounds such as polysaccharides, proteins, fatty acids, and phenols. Calculated on dry weight, SCG comprise as much as 66% of polysaccharides, mostly galactomannans (50%), cellulose (25%), and arabinogalactans (25%). In particular, galactomannans seem to be a suitable raw material for edible and biodegradable foil production, because they are characterized by good solubility in water and can form very viscous and stable aqueous solutions with film-forming capacity. In addition, galactomannans are stable at temperatures ≤200 °C, even during prolonged exposure (>3 h) [12]. The chemical composition of SCG is rich in phenolic components, mainly chlorogenic acids (85%) such as 4-O-caffeoylquinic acid, 5-O-caffeoylquinic acid, and 3-O-caffeoylquinic acid, and also caffeic acid (6%) [6]. Moreover, SCG have a high protein content (up to 17%) [13] and lipids (13–15%), a majority of which is coffee oil [14]. The coffee oil contains more than 40% linoleic acid, and thus has ultraviolet absorption properties, making it suitable for cosmetics production. Moreover, the unsaponified part (19%) of coffee oil contains plenty of biologically active compounds, including sterols, tocopherols, phosphatides, and free diterpenes (cafestol and kahweol), which endow coffee oil with biological activities such as antioxidant, antibacterial, antiproliferative, and anti-inflammatory properties. Since SCG contain about 20% extractable oil, they can easily be adapted to produce edible/biodegradable film for food packaging. The oil chemical composition of SCG is rich in natural polymers such as polysaccharides, which may improve the mechanical parameters of the film texture. In addition, the antioxidant properties of SCG oil make it a functional packaging additive that can prolong the shelf-life of food [15]. 

Of the 130 species in the *Coffea genus* species known to science, *Coffea arabica* (commercially known as Arabica) and *Coffea canephora* (commercially known as Robusta) represent the most widely cultivated and consumed coffee plant species worldwide [15]. Currently, approximately 60% of the coffee produced and marketed in the world comes from Arabica plants, and nearly 40% is obtained from Robusta crops [16]. Since Robusta coffee and Arabica coffee belong to different species, their physical and chemical characteristics differ significantly, and their oil fraction also shows differences in chemical composition [17]. In general, Arabica coffee grains contain more of the lipid trigonelline; on the other hand, Robusta grains have higher caffeine and chlorogenic acids concentrations [18]. Furthermore, the kahweol concentration is higher in Arabica coffee oil (ACO), while 16-O-methyl cafestol is abundant in Robusta coffee oil (RCO) [19,20]. The differences in chemical composition between ACO and RCO may translate into different effects on edible/packaging film properties; therefore, an individual approach to the study of their properties is necessary. 

This study presents the possibility of using coffee grounds for production of edible/biodegradable packaging. ACO/RCO was used to prepare emulsions that were incorporated into κ-carrageenan film-forming solution. Further analysis was conducted to evaluate how the addition of ACO/RCO affects the physicochemical and textural properties of experimentally produced packaging. 

Films based on polysaccharides are promising alternatives to petrol-derived plastic films. κ-carrageenan is considered an excellent naturally occurring material for food packaging coatings due to its unique film-forming ability. In addition, the American Food Drug Administration (FDA) confirms the safety of carrageenan and allows its use as a food ingredient [21]. 

## 2. Materials and Methods

### 2.1. Extraction of the Oil from Spent Coffee Grounds 

The spent coffee grounds used for oil extraction came from a mobile coffee shop in Brno (Czech Republic), which consisted of a blend of Arabica coffee (originating from Brazil) and Robusta coffee (originating from Vietnam). The Arabica to Robusta coffee mixture ratio was 50:50. Spent coffee grounds were also sampled from a co-located coffee shop (Brno, Czech Republic) that consisted only of Arabica coffee (Brazil origin). For the extraction of the oil, we weighed 200 g of ground coffee from each type of sample and mixed it with 400 mL of hexane. The mixture of coffee and hexane (Avantor Performance Materials Poland) was allowed to stand for 5 days, and then it was filtered through filter paper. The hexane residues were evaporated from the solution by stirring at 250 rpm for 4 h, without the application of heat. The obtained coffee oil samples were stored in a refrigerator.

### 2.2. Emulsion Preparation

To prepare 100 mL of emulsion, 82.5 mL of _d_H_2_O, 15 g of coffee oil, and 2.5 g of liquid sunflower lecithin (Roth) were used. Emulsification was performed using a Pulse 150 Ultrasonic Homogenizer (Benchmark, Sayreville, NJ, USA). 

### 2.3. Edible/Biodegradable Package Preparation

**Figure 1:**
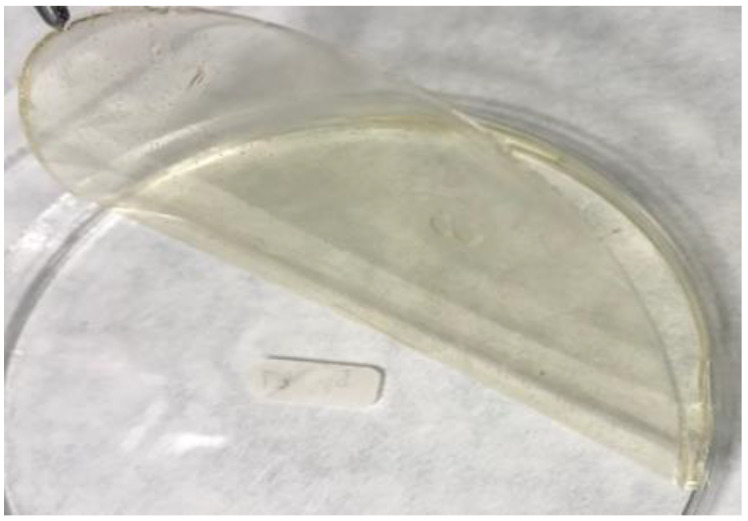
Example of a film prepared with the 1 g coffee emulsion addition.

Each of the edible/biodegradable films was prepared using 0.3 g of κ-carrageenan powder. The packaging sheets with the addition of coffee emulsion contained 0.1, 0.45, 0.8, or 1 g of this emulsion. The control sample (CC) did not contain coffee emulsion additives. Distilled water was added to a final volume of 45 mL (Table 1). The samples were stirred at 50 °C until dissolution was complete. Subsequently, 250 µL of glycerol (Lach-Ner, Neratovice, the Czech Republic) was added to the dissolved mixture, followed by 5 min of stirring. After the elapsed time, 1.5 mL of Tween 80 was added to the mixture and stirred for 7 min. The resulting mixture was poured into the lids of Petri dishes (Anicrin, Scorze, Italy) with a diameter of 9 cm and allowed to solidify. An example of the experimentally produced film is shown in Figure 1.

### 2.4. Determination of Fat Content in Spent Coffee Grounds

The total fat content was obtained with the Soxhlet method, using the B-811 apparatus from Büchi (Flawil, Switzerland). The extraction was carried out using 5 g of spent coffee grounds, with petroleum ether (Lach-Ner) as a solvent. The program consisted of 90 min of extraction followed by 30 min of washing, and followed by 20 min of solvent evaporation.

### 2.5. Determination of Acid Value (AV) in Oil Extracted from Spent Coffee Grounds

The evaluation of the acidity value was carried out following ISO 660:2009. Briefly, 50 mL of diethyl ether (VWR International Belgium) was added to 5 g of the extracted coffee oil, and 1 mL of 1% phenolphthalein (Lach-Ner) was used as an indicator. The solution was mixed for one minute, and the sample was titrated with 0.1 M KOH (Lach-Ner). 

### 2.6. Determination of Peroxide Value (PV) in Oil Extracted from Spent Coffee Grounds 

The peroxide number was estimated according to the requirements of ISO 3960:2017. Briefly, 5 g of coffee oil samples was added into 30 mL of a solution of glacial acetic acid (Lach-Ner) and chloroform (Penta s.r.o., Prague, the Czech Republic) (2:3). The mixture was shaken gently for 1 min. Then, 30 mL of _d_H_2_O and 5 mL of 1% starch (Penta s.r.o.) solution were added. Subsequently, 0.01 M Na_2_S_2_O_3_ (Penta s.r.o.) was used for sample titration. Instead of a sample, the _d_H_2_O was used as a blank.

### 2.7. Determination of Total Polyphenols 

The polyphenol content was analyzed via the Folin–Ciocalteau method according to the protocol described by Tomadoni et al. [22]. In brief, 20 mL of an ethanol (VWR International France)–water solution (1:1) was poured into a dark glass vial containing exactly 0.1 g of the edible packaging sample. The samples were extracted in an ultrasonic bath for 30 min. Subsequently, 1 mL of an extract was transferred into a 25 mL volumetric flask and mixed with 5 mL of Folin–Ciocalteau (Penta sro) solution (diluted 1:10) and 4 mL of 7.5% Na_2_CO_3_ (Lach-Ner). The samples were mixed vigorously and left in the dark for 30 min. The absorbance was recorded at 765 nm. As a blank, 1 mL of _d_H_2_O was used instead of the sample. The obtained results are presented as mg of gallic acid per gram of sample.

### 2.8. Determination of Antioxidant Capacity; FRAP (Ferric Reducing Antioxidant Power)

In this part of the experiment, the method proposed by Behbahani et al. was implemented [23]. At first, a FRAP working solution was prepared by mixing 300 mM acetate buffer (pH 3.6) with 10 mM TPTZ (Sigma-Aldrich, St. Louis, MO, USA) solution (dissolved in 40 mM HCl (Dr. Kulich Pharma s.r.o., Hradec Králové, the Czech Republic), and 20 mM FeCl_3_ (Sigma-Aldrich) solution (in the ratio of 10:1:1). The samples for analysis were prepared in dark tubes. Analyzed samples contained 180 µL of an extract, 300 µL of _d_H_2_O, and 3.6 mL of working solution. All samples were left in the dark for 8 min. Then, the absorbance was collected at 593 nm against a blank (FRAP working solution and _d_H_2_O). The calibration curve was constructed using the Trolox (Sigma-Aldrich) dilution series, thus the results are expressed as µmol of Trolox per one gram of sample.

### 2.9. Determination of Antioxidant Capacity; ABTS (2,2′-Azinobis (3-ethylbenzothiazoline-6-sulfonic Acid))

The antioxidant activity was determined by the ABTS method according to the protocol described by Thaipong et al. [24]. Approximately 12–16 h before the measurement, 10 mL of 0.007 M ABTS (Sigma-Aldrich) was mixed with 10 mL of 0.00245 M potassium persulfate (Lach-Ner) solution, and the mixture was allowed to react. Then, the prepared ABTS solution was diluted with ethanol until an absorbance of 0.7 at 734 nm was reached. Extracts for this analysis were prepared in the same way for both sample types, coffee oil, and coffee oil-spiked emulsion. Each extract was obtained by adding 20 mL of ethanol–water (1:1) solution to 0.1 g of sample, then the samples were sonicated for 30 min and filtered. For measurement, 20 μL of extract was added to 1980 μL of ABTS solution and the mixture was kept aside for 5 min in the dark. The absorbance of samples including the blind sample was recorded at 734 nm. The percentage inhibition was estimated using the following pattern:

ABTS (%) = (A0 − A1)/A0 × 100%

### 2.10. Determination of Antioxidant Capacity; CUPRAC (Cupric Ion Reducing Antioxidant Capacity)

The antioxidant activity was also evaluated using the CUPRAC technique, and this analysis was performed according to the protocol of Apak et al. [25]. Each extract was obtained by adding 20 mL of ethanol–water (1:1) solution to 0.1 g of sample, then the samples were sonicated for 30 min and filtered. A mixture subjected to analysis was prepared by mixing 1 mL of 0.01 M copper (Sigma-Aldrich), 1 mL of 0.0075 M neuocuproine (Sigma-Aldrich), 1 mL of NH_4_Ac (ammonium acetate) buffer pH = 7.0, 0.1 mL of ethanol–water solution (1:1), and 1 mL of extract. The absorbance was measured against a blank at 450 nm after 1-hour incubation in the dark. A dilution series of Trolox solution was used to generate the calibration curve, and the output results are expressed as µmol of Trolox per gram of sample.

### 2.11. Determination of Malondialdehyde Content (MDA)

The MDA assessment was performed with the thiobarbituric acid (TBA) method according to the previously published methodology [26]. At first, 1.5 g of sample was weighed and placed in a centrifuge tube; then, 1 mL of EDTA (Penta s.r.o.) and 5 mL of 0.8% BHT (Acros organics) were successively added and mixed. Subsequently, TCA (Lach-Ner) solution (10%) was added to the mixture, and the samples underwent homogenization for 30 s at 10,000 rpm. After homogenization, the tube was centrifuged for 5 min at 3500× *g* at 4 °C. The lower layer was collected and then filtered. The filtrate was transferred to a volumetric flask and filled to a volume of 10 mL with 10% TCA solution. Furthermore, 4 mL of the mixture was transferred to a new flask, 1 mL of TBA (Sigma-Aldrich) was added, and the mixtures were incubated for 90 min at 70 °C. After incubation, the samples were kept in an ice bath for 2–3 min and then left at room temperature for 45 min. The absorbance was recorded at 532 nm. The standard calibration curve was used to estimate the results.

### 2.12. Textural Properties

The tensile strength (TS) and elongation at break (EB) were measured according to ASTM International Test Method ASTM D882-02, using a TA.XT plus texture analyzer (Godalming, UK). The prepared edible packages were cut into 1 × 5 cm rectangles, and each measurement was repeated five times.

### 2.13. Water Content, Solubility, and Swelling Degree

The analysis was conducted according to the protocol described by Souza and colleagues, with minor modifications. The packaging samples were cut into 2 × 2 cm squares and then weighed on an analytical scale (KERN, Balingen, Germany); the mass of the samples was marked as W1. Subsequently, the samples were placed for 2 h in an oven (Ecoccel 55) set to 105 °C and then weighed again (W2). Subsequently, the samples were placed in _d_H_2_O or seawater and then dried at room temperature for 24 h and weighed again (W3). After incubation at RT, the samples were placed in an oven heated to 105 °C for 24 h and then weighed again (W4). Six replicates were prepared for each foil sample. The solubility was measured in _d_H_2_O and seawater from the Gdańsk quay (Gdańsk, Poland) under map coordinates: 54.342504, 19.028122. The following equations were used for calculation:

Swelling degree (%) = [(W3 − W2)/W2] × 100

Solubility (%) = [(W2−W4)/W2] × 100

### 2.14. Statistical Analysis

One-way ANOVA, parametric Tukey’s post hoc test (in case Levene’s test showed equal variances, *p* > 0.05), and non-parametric Games–Howel post hoc test (in case Levene’s test showed unequal variances, *p* < 0.05) were used to evaluate statistical significance at the *p* < 0.05 level for differences within groups. Statistical analysis was performed using the program SPSS 20 (IBM, Corporation, Armonk, NY, USA).

## 3. Results and Discussion

In the first phase of the experiment, the chemical properties of oils extracted from SCG obtained from Arabica coffee beans and an Arabica/Robusta coffee blend (in a 50:50 ratio) were determined. Table 2 presents the results of the analyses performed to assess the degrees of oxidation and hydrolysis of SCG oils and their antioxidant capacities. The results obtained for 100% Arabica coffee oil (ACO) and Arabica/Robusta coffee oil (ACO/RCO) were evaluated according to Codex Alimentarius quality standards and compared with results available in the scientific data repositories. 

The PV, MDA, and AV markers were estimated for both types of SCG oils, as these parameters are crucial for the determination of quality and the safety of the edible fats, including coffee oil. For example, a high PV indicates the presence of primary products of lipid oxidation, such as lipid hydroperoxides, which are formed during the initial rancidity of the oil [27,28]. Thereby, a high peroxide value indicates lower oil stability and its susceptibility to fast degradation. In the present study, the PVs for ACO and ACO/RCO were below the Codex cut-off value, which is set at 15 meqO_2_/kg [29] and were 5.01 meqO_2_/kg and 4.11 meqO_2_/kg, respectively. Our results fall within the range of values measured in other independent studies. In a study performed by Panpraneecharoen and colleagues, the PV of an ACO extracted with 100% n-hexane reached 23.96 ± 2.09 meqO_2_/kg and was even higher than the value reported in our study, despite using the same solvent [30]. Subsequently, in the oil extracted from Arabica/Robusta SCGs, the PV was as high as 5.21 ± 0.1 meqO_2_/kg in the study performed by Bijla and collective [31]. The primary products of lipid oxidation are gradually converted to secondary breakdown products such as aldehydes, ketones, carbonyls, trienes, and malondialdehyde, among others [32]. These by-products of fat oxidation give a rancid, unpleasant taste and are harmful to humans. The use of rancid oil as an additive in the production of edible/packaging films may impair the taste and quality of the wrapped food. Furthermore, the rancid oil contained in the packaging film can also accelerate food spoilage [33]. Malondialdehyde (MDA) is one of the secondary lipid oxidation products; it is abundant and stable and represents one of the main indicators of the lipid oxidation state [34,35,36]. Due to the high reactivity of MDA, it has been recognized as a cytotoxic, neurotoxic, and mutagenic compound. The European Food Safety Authority Scientific Committee suggests an MDA exposure level of 30 μg per one kg of body weight per day as a toxicological hazard threshold [37]. Cong et al. stated that food products with a TBA concentration of <0.576 mg MDA/kg are considered fresh; those with a TBA value of 0.65–1.44 mg MDA/kg are rancid but still within acceptable limits; and those with a TBA concentration >1.5 mg MDA/kg are rancid and unsuitable for consumption [38]. In the study performed by Viau et al. [39], the formation of MDA in various edible oils during storage varied from 43.2 to 79.2 μg/g. Moreover, in the same paper, they reported that MDA did not correlate with PV levels, and MDA was measurable in fresh oils whose PV and AV values were below the recommended limits for edible oils [40]. The MDA values in our study were within acceptable limits; however, the oil isolated from the SCG of the 50/50 Arabica/Robusta coffee blend showed a much more satisfactory quality, as the MDA concentration was 0.5 ± 0.02 µg/g. This value indicates that the ACO/RCO oil is of very good quality and does not undergo rancidification. On the other hand, 100% ACO oil still remains within the limit; however, based on the results of Bouyanfifa et al. [41], the value of 0.73 ± 0.1 µg/g may indicate an early stage of oil rancidity. Similar results were obtained by Muangrat et al. [42]; ACO oil extracted from SCG (Soxhlet and accelerated solvent extraction, propanol used as a solvent, and supercritical CO_2_ extraction methods) showed a TBA value of 0.76 mg MDA/kg. In addition, we noticed that the acidity value of the ACO samples reached 7.31 mg KOH/g, which is consistent with the results obtained by extraction of SPG oil with n-hexane in other independent studies [42,43,44,45]. The AV of the ACO/RCO samples was higher, and it was 9.09 mg KOH/g, but there was no significant difference. 

The antioxidant capacity of both SPG oils was determined to assess the suitability of coffee oil for the subsequent production of edible/biodegradable packaging films. Coffee beans contain many compounds with antioxidant activity, such as phenolic compounds, and chlorogenic, ferulic, and caffeic acids. Among the polyphenols, chlorogenic and gallic acid effectively inhibit oxidation by eliminating reactive oxygen species induced by lipid peroxidation. In addition, caffeine and trigonelline demonstrated antiradical activities in in vitro and in vivo analyses. It was confirmed that Maillard reaction products predominate in roasted coffee beans, which contribute to antioxidant activity [46]. The association between the antioxidant capacity of coffee brews to the coffee beans’ roasting degree was also intensively analyzed [47,48,49]. Further, it has been proven that after the brewing process, a significant part of bioactive compounds and antioxidants remains in the spent coffee [50]. For example, Choi et al. [51] showed that more than 95% of the DPPH activity was retained in the spent coffee referring to the roasted coffee. Recent research reported that utilization of by-products such as SCG can provide the oil with high quality and also contain valuable components such as essential fatty acids or substances with antimicrobial activity [52]. The ACO and ACO/RCO investigated in our study showed significantly high antioxidant potential (Table 2), comparable to cold-pressed vegetable oils including canola oil and olive oil [53,54]. These results confirm that SCG oil can serve as a substrate with excellent antioxidant properties, which can positively affect the functional characteristics of the edible/biodegradable film. Further, it was determined whether there was a visible difference between the oil extracted from used coffee grounds that contained a mixture of Arabica and Robusta coffee varieties and coffee grounds that contained only the variety Arabica coffee. Based on the results, it appears that ACO/RCO is superior to ACO in terms of antioxidant capacity, as ACO/RCO performed better in FRAP and ABTS analyses (128.71 Trolox µmol/g and 10.84%, respectively), while ACO achieved 118.27 Trolox µmol/g and 7.45%, respectively. The better antioxidant properties of ACO/RCO can be explained by differences in the biochemistry of Arabica and Robusta cultivars. The study performed by Song et al. noted that compared to Arabica coffee, Robusta is characterized by a higher concentration of bioactive compounds and overall antioxidant activity [55]. In particular, the amount of caffeine is usually quoted as twice the concentration of caffeine measured in Arabica coffee [56,57,58]. The incorporation of ACO/RCO into a ĸ-carrageenan-based film can provide the formation of the active polymer from which active antioxidant molecules will gradually migrate toward food. Consequently, this can delay food spoilage and extend the shelf-life of packaged food. Therefore, we assumed that the addition of ACO/RCO in biodegradable films based on κ-carrageenan would be the most optimal for this study. 

The antioxidant activity and total polyphenols contents of edible/biodegradable films are presented in Table 3.

Based on the results, it has been proven that κ-carrageenan-based films are capable carriers of SCG-derived phenolic compounds, as their total polyphenols content ranges from 3.11 to 3.85 mg gallic acid/g. In the control sample (CC), the polyphenols were also measurable but in negligible amounts (0.11 mg gallic acid/g) in comparison to the films with ACO/RCO emulsions. This observation is correct and consistent with the finding of Souza et al., who reported that polysaccharides derived from red algae (such as carrageenan) exhibit antioxidant activity, and this activity correlates with the number of sulfate groups [59]. Nevertheless, the difference in TP concentration between biofilms prepared with ACO/RCO emulsions is much greater than in the case of the film without the addition of ACO/RCO. The final concentration of the ACO/RCO in the edible films was low, since for film-forming we used oil in emulsion form; nevertheless, our results closely correspond to those obtained in studies using pure SCG oil for edible foil preparation. For instance, Lombo Vidal et al. presented results in which a biodegradable film prepared with 0.2% coffee oil showed a TP concentration of 1.3 mg/g to 5.44 mg/g [60]. Most recently, Dordevic and collective [61] published results of the experiment in which he compared the properties of biofilms comprising ACO obtained from SCG, using the Tween20 or Tween 80 as emulsifiers. The total polyphenols concentration for biofilms produced with Tween 80 ranged from 3.55 to 7.00 mg gallic acid/g, while for Tween 20 the values ranged from 2.23 to 3.80 mg gallic acid/g. The antioxidant capacity, attributed to the presence of polyphenolic compounds, increased compared to the control film samples (prepared without the addition of SCG oil emulsion). This increase was most significant when measured by the CUPRAC method, likely due to its ability to effectively react with both hydrophilic and lipophilic antioxidants. The ABTS method also showed an increase, reflecting its sensitivity to both hydrophilic and lipophilic antioxidants, though to a lesser extent. The FRAP method, which measures the ability to reduce ferric ions, indicated a moderate increase, as it primarily detects hydrophilic antioxidants. These differences highlight the varied responses of each method to the specific antioxidant properties present in the samples.

The mechanical properties of edible packaging films are typically determined by their tensile strength and elongation at break [62]. These parameters are crucial to determine the suitability of the packaging material for processing in the entire technological process, including printing, laminating, and packaging, as well as further resistance during transport, handling, and storage [63]. In the context of materials science, the tensile strength (MPa) of an edible film represents the maximum stress the film can withstand before breaking. In terms of elongation at break (%), its value indicates the film’s ability to withstand shape changes without cracking [64,65,66]. Throughout the life cycle of a packaging material, it is exposed to many external forces, such as pulling and stretching, which is why their thresholds are an important decision-making tool in the entire process of designing and manufacturing edible films intended for a specific application. The mechanical properties of experimentally produced edible/degradable films are presented in Table 4. 

The addition of the ACO/RCO emulsion significantly affected the tensile strength of ACO/RCO biofilms. The addition of 0.1 mL of the ACO/RCO emulsion resulted in TS reduction of the CC01 sample by more than half compared to the control CC sample (0.053 MPa, 0.121 MPa, respectively). The same effect was described in the latest report [61], where κ-carrageenan-based edible film with 0.1 mL of SPG oil added showed a TS of 0.06 MPa, while the control sample had a TS of 0.13 MPa. On the other hand, based on recent reports, the coffee oil may have a different effect on the ST of the edible biofilm, depending on the base polymer that was used to produce the film. For example, the TS of carboxymethyl cellulose-based films was not significantly worsened by the addition of green coffee oil [60]. Furthermore, the corn starch-based films blended with green Robusta coffee oil exhibited higher tensile strength in comparison to pure corn starch film [14]. However, in the same study, the highest concentration of oil did not reveal the best textural properties in terms of tensile strength. The similarity of this mechanism is visible in our study, where the CC1 sample with the highest concentration of ACO/RCO showed the lowest TS compared to the samples with a lower content of ACO/RCO. This phenomenon may occur because the plasticizer present in high concentration increases the mobility of the polysaccharide matrix by increasing the intermolecular spacing and reducing the intermolecular interaction of the carrageenan. These plasticizers, such as coffee oil, enhance chain mobility by distorting hydrogen bonding between the neighboring polymer chains [67]. Interestingly, the elongation at break significantly increased (*p* < 0.05) in κ-carrageenan-based films blended with the ACO/RCO emulsion. The CC01 sample showed the highest level of EB and was 62.4% higher than the CC sample; the EBs reached 123.377% and 77.03%, respectively. The corn starch-based films with 0.25% RCO showed an EB of 35.12%, which is almost half the value of sample CC1 (69.609%), which contains 0.33% ACO/RCO. Moreover, the edible biofilms in our study with lower ACO/RCO concentrations (CC01, CC045, CC08) showed higher elasticity than corn starch-based films with RCO concentrations of 0.5% and 1% (42.30% and 29.10%, respectively) [14]. Polysaccharide films like κ-carrageenan may be too brittle due to the neat structure of κ-carrageenan, which is not necessarily desirable for packaging materials. Therefore, the plasticization of the raw polysaccharide-based package film is important to improve their rigidity and stiffness to enlarge the versatility of the edible foils/films/coats. The ability of a plasticizer to form an appropriate film or coating depends on its compatibility with κ-carrageenan. SCG oil seems to be a suitable plasticizer due to the presence of free hydroxyl groups, which reduces the brittleness of κ-carrageenan [68]. The value of the film’s EB in the presented study could have been influenced by the addition of glycerol, which is often used as a plasticizer for casting edible films [69,70,71,72]. Nevertheless, all of the edible films were produced with the addition of the same amount of glycerol; therefore, the potential effect of SCG on EB is trackable. For edible films, a high EB is preferred because it makes films more elastic and less easy to tear; therefore, a high EB gives the films a better ability to envelop and wrap the food products [65]. 

The water affinity results, such as water content (WC), water solubility in both distilled water and seawater, and swelling degree in seawater of edible/biodegradable packages, are presented in Table 5. 

The water content of the pure κ-carrageenan film was the highest (29.79% ± 1.34), and the water content of the edible films with emulsified ACO/RCO was significantly lower (*p* < 0.05). The introduction of a 0.1 mL ACO/RCO emulsion to the κ-carrageenan film casting protocol resulted in a decrease in the water content by more than half in the CC01 sample (11.04%), while the WC reduction was as much as 80% in the experimentally prepared biofilm containing the 0.8 mL ACO/RCO emulsion (6.01% ± 1.69). Coffee oil contains unsaponifiable substances that have a hydrophobic effect and reduce affinity for water molecules, resulting in a decrease in moisture content of κ-carrageenan-based films. In general, edible films with a low water content are preferred, otherwise, their use as direct food packaging may result in water ingress into the product, which may result in the deterioration of product quality and shortening its shelf-life [73]. Furthermore, we observed that the solubility of the edible/biodegradable films in distilled water did not differ between the edible/biodegradable films prepared with the ACO/RCO emulsion and those without the addition of emulsified oil. The solubility found after 24 h of immersion of the film in distilled water was 100% for all types of κ-carrageenan biofilms. All biofilms with the addition of the ACO/RCO emulsion also showed 100% solubility in seawater, while the solubility of the control sample was 45.95%. However, in another study, complete dissolution of carrageenan-based biofilms was observed after seven days [74]. Water solubility is an important parameter indicating the susceptibility of a biofilm to maintain integrity in aqueous systems [75]. The solubility of the edible/biodegradable film is largely contingent on the nature of the film’s base material. The κ-carrageenan solubility rate is high and is influenced by multiple factors, such as the sulfate group on the molecule and the content of 3, 6-anhydrogalactose [76]. Therefore, it is anticipated that the inclusion of hydrophobic components such as oil in the carrageenan matrix may enhance the biofilm’s surface hydrophobicity as a result of the formation of a strong stabilizing network crosslink via covalent and non-covalent bonds [77]. On the other hand, it has been shown that the application of plasticizers into biofilm production may promote an increase in the solubilization of the films. There are indications that the plasticizer used may detach from the polymer matrix, causing the formation of cavities in the material and making it more accessible to water molecules [78]. The high solubility of packaging materials is desired when it is intended to be used as edible packaging. These films also have good biodegradability; however, due to their low water resistance, they are not recommended for wrapping food with high moisture content. For high water-content food applications, films that are not dissolvable in water are a prerequisite. However, for ecological reasons, we aimed in this study to develop a packaging material that is worth eating and is also characterized by high water solubility [79].

## 4. Conclusions

In this research, we successfully developed κ-carrageenan-based packaging enriched in emulsified SCG oil. The oil pressed from a blend of Robusta and Arabica coffee was selected for experimentation, since it revealed the best quality and the highest antioxidant properties in comparison to the ACO alone. The packaging showed high biodegradability and ACO/RCO oil emulsion; additionally, the emulsion provided antioxidant compounds, which gave the films active film properties. Analysis showed that the ACO/RCO enhanced the level of polyphenols in the films, and the antioxidant capacity of the films also increased significantly, as shown in the CUPRAC test. Moreover, we noticed that ACO/RCO performed as a good plasticizer for κ-carrageenan, reducing its brittleness and enhancing the elongation at the break of the films. The films demonstrated 100% solubility, ensuring full biodegradability and minimizing environmental impact while being potentially suitable for various packaging applications. SCG enhance the films’ flexibility and antioxidant properties, making them a possible sustainable and functional alternative for the food packaging industry.

## Figures and Tables

**Table 1:** Descriptions and contents of samples.

Sample ID	Coffee Emulsion Addition (g)	Ingredients
CC	0	Carrageenan + glycerol +Tween 80 + _d_H_2_O
CC01	0.1	Carrageenan + coffee emulsion + glycerol + Tween 80 + _d_H_2_O
CC045	0.45	Carrageenan + coffee emulsion + glycerol + Tween 80 + _d_H_2_O
CC08	0.8	Carrageenan + coffee emulsion + glycerol + Tween 80 + _d_H_2_O
CC1	1	Carrageenan + coffee emulsion + glycerol + Tween 80 + _d_H_2_O

**Table 2:** Chemical characteristics of 100% ACO and ACO/RCO (50/50 coffee blend) isolated from SCG.

Analysis	100% ACO	50/50 ACO/RCO
Peroxide value (meqO_2_/kg)	5.01 ± 0.78	4.11 ± 0.48
Malondialdehyde (µg/g)	0.73 ± 0.1 ^b^	0.50 ± 0.02 ^a^
Acid value (mg KOH/g)	7.31 ± 1.00	9.09 ± 0.39
FRAP (Trolox µmol/g)	118.27 ± 7.39 ^b^	128.71 ± 5.70 ^a^
ABTS (%)	7.45 ± 0.34 ^b^	10.84 ± 0.49 ^a^
CUPRAC (Trolox µmol/g)	117.22 ± 5.07	N/A
Total polyphenols (mg gallic acid/g)	14.87 ± 0.06	13.96 ± 2.03

Lowercase letters in superscript indicate a statistically significant difference (*p* < 0.05) in the same row.

**Table 3:** Antioxidant profiles and total polyphenols contents of edible/biodegradable films.

Sample ID	FRAP (Trolox µmol/g)	ABTS (%)	CUPRAC (Trolox µmol/g)	Total Polyphenols (mg gallic acid/g)	Malondialdehyde (µg/g)
CC	3.09 ± 0.39	4.52 ± 1.00	4.71 ± 0.72 ^a^	0.11 ± 0.02 ^a^	0.58 ± 0.14 ^a^
CC01	2.79 ± 0.09 ^a^	3.94 ± 0.19	11.77 ± 4.38	3.25 ± 0.09 ^b^	6.81 ± 0.21 ^c^
CC045	3.37 ± 0.23 ^b^	4.37 ± 0.77	15.38 ± 0.83 ^b^	3.11 ± 0.03 ^b^	7.04 ± 0.79d ^c^
CC08	3.33 ± 0.21 ^b^	4.33 ± 0	22.04 ± 0.77 ^c^	3.85 ± 0.13 ^c^	23.85 ± 2.72 ^b^
CC1	3.21 ± 0.15 ^b^	4.37 ± 0.01	23.90 ± 1.23 ^dc^	3.41 ± 0.17 ^b^	19.53 ± 0.30 ^b^

Lowercase letters in superscript indicate a statistically significant difference (*p* < 0.05) in the same column.

**Table 4:** Textural parameters of the edible/biodegradable films.

Sample ID	Tensile Strength (MPa)	Elongation at Break (%)
CC	0.121 ± 0.023	77.03 ± 13.264
CC01	0.074 ± 0.013	84.314 ± 4.155
CC045	0.063 ± 0.01	90.336 ± 2.235
CC08	0.071 ± 0.007	90.336 ± 2.235
CC1	0.074 ± 0.026	84.880 ± 1.321

**Table 5:** Solubilities of the edible/biodegradable films in distilled water and seawater.

Sample ID	Solubility in _d_H_2_O (%)	Solubility in Seawater (%)	Swelling Degree in Seawater (100%)	Water Content– _d_H_2_O
CC	100	45.95 ± 27.67	383.88 ± 32.77	29.79 ± 1.34 ^a^
CC01	100	100	-	11.04 ± 3.57 ^bc^
CC045	100	100	-	7.98 ± 0.19 ^c^
CC08	100	100	-	6.01 ± 1.69 ^bc^
CC1	100	100	-	6.72 ± 0.32 ^b^

Lowercase letters in superscript indicate a statistically significant difference (*p* < 0.05) in the same column.

## Data Availability

The original contributions presented in the study are included in the article, further inquiries can be directed to the corresponding author.
